# Element Abundances: A New Diagnostic for the Solar Wind

**DOI:** 10.3847/1538-4357/ab23f1

**Published:** 2019-07-15

**Authors:** J. Martin Laming, Angelos Vourlidas, Clarence Korendyke, Damien Chua, Steven R. Cranmer, Yuan-Kuen Ko, Natsuha Kuroda, Elena Provornikova, John C. Raymond, Nour-Eddine Raouafi, Leonard Strachan, Samuel Tun-Beltran, Micah Weberg, Brian E. Wood

**Affiliations:** 1 Space Science Division, Code 7684, Naval Research Laboratory, Washington, DC 20375, USA;; 2 Johns Hopkins University Applied Physics Laboratory, Laurel. MD 20723, USA; 3 Space Science Division, Code 7686, Naval Research Laboratory, Washington, DC 20375, USA; 4 Department of Astrophysical and Planetary Sciences, Laboratory for Atmospheric and Space Physics, University of Colorado, Boulder, CO 80309, USA; 5 University Corporation for Atmospheric Research (UCAR), Boulder, CO 80307, USA, and Space Science Division, Code 7684, Naval Research Laboratory, Washington DC 20375, USA; 6 Smithsonian Astrophysical Observatory, Cambridge, MA 02138, USA; 7 NRL/NRC Research Associate, Space Science Division, Code 7684, Naval Research Laboratory, Washington, DC 20375, USA; 8 Space Science Division, Code 7685, Naval Research Laboratory, Washington, DC 20375, USA

**Keywords:** solar wind, Sun: abundances, Sun: chromosphere, turbulence, waves

## Abstract

We examine the different element abundances exhibited by the closed loop solar corona and the slow speed solar wind. Both are subject to the first ionization potential (FIP) effect, the enhancement in coronal abundance of elements with FIP below 10 eV (e.g., Mg, Si, Fe) with respect to high-FIP elements (e.g., O, Ne, Ar), but with subtle differences. Intermediate elements, S, P, and C, with FIP just above 10 eV, behave as high-FIP elements in closed loops, but are fractionated more like low-FIP elements in the solar wind. On the basis of FIP fractionation by the ponderomotive force in the chromosphere, we discuss fractionation scenarios where this difference might originate. Fractionation low in the chromosphere where hydrogen is neutral enhances the S, P, and C abundances. This arises with nonresonant waves, which are ubiquitous in open field regions, and is also stronger with torsional Alfvén waves, as opposed to shear (i.e., planar) waves. We discuss the bearing these findings have on models of interchange reconnection as the source of the slow speed solar wind. The outflowing solar wind must ultimately be a mixture of the plasma in the originally open and closed fields, and the proportions and degree of mixing should depend on details of the reconnection process. We also describe novel diagnostics in ultraviolet and extreme ultraviolet spectroscopy now available with these new insights, with the prospect of investigating slow speed solar wind origins and the contribution of interchange reconnection by remote sensing.

## 1. Introduction

The prediction of the existence of the solar wind (Parker 1958) must rank as one of the key theoretical insights in the history of heliophysics. Since its discovery (Gringauz et al. 1960; Neugebauer & Snyder 1962), Parker’s original concept of a wind driven by thermal pressure in a corona heated by magnetohydrodynamic (MHD) waves (Parker 1963) has been slightly modified to a scenario where the MHD waves drive the wind directly (e.g., Belcher 1971; Isenberg & Hollweg 1982; Ofman 2010). The fast solar wind is established to emerge from coronal holes, open field regions where plasma emerges directly from the solar chromosphere into the wind, and exhibits largely unbalanced Alfvénic turbulence (Bruno & Carbone 2013; Ko et al. 2018). By contrast the slow solar wind, which shows strong chemical fractionation effects in its composition and more balanced (or lower cross-helicity) turbulence, is frequently believed to originate in closed coronal loops where the fractionation occurs (e.g., Antiochos et al. 2011), before being released into the solar wind by interchange reconnection with the surrounding open field, as well as possibly coming directly from the open field like the fast solar wind (Cranmer et al. 2007).

Solar wind acceleration and composition depend on processes at three transition layers in the solar upper atmosphere. The first, usually located in the low chromosphere, is where the pressure changes from being thermally dominated to being magnetically dominated. In this region the sound speed and Alfvén speed are equal, and a number of processes involving wave mode conversion and other wave–wave interactions can occur. This is where a significant fraction of the MHD waves that eventually accelerate the solar wind are generated from motions ultimately deriving from solar convection. The second transition layer appears higher up in the chromosphere, where largely neutral gas gives way to the ionized plasma that ends up as the solar corona and wind. This transition gives rise to strong density gradients and associated wave reflection and refraction. Alfvén waves interacting with this density gradient generate the ponderomotive force. This combines the effect of the wave pressure gradient and the force on the plasma wave due to wave reflection and refraction. Since the waves are fundamentally magnetic in character, only ions see this force, and ion–neutral separation is the result, giving rise to element fractionation in the upper atmosphere known as the first ionization potential (FIP) effect. This abundance anomaly has been seen in the solar corona and wind for over 50 years (e.g., Pottasch 1963), and can be seen to offer a key observable for wave processes that until now has remained largely unexploited.

The third transition layer, and arguably the hardest to understand in a quantitative theoretical manner, is the evolution of the solar plasma from a fluid to a collisionless plasma dominated by kinetic effects. This happens where the ion– proton collision rate becomes slower than the solar wind expansion rate, *v*_*w*_(*r*)/*r*, where *v*_*w*_(*r*) is the solar wind speed and *r* is the heliocentric radius. With the ion–proton collision rate at freeze-in given by

(1) $$\nu_{ip}=\frac{4\pi e^{4}\text{ ln}\Lambda }{m_{p}}\frac{n}{k_{\text{B}}T}\frac{Z^{2}}{A+1}\frac{4}{3\sqrt{\pi }}\sqrt{\frac{A}{A+1}\frac{m_{p}}{2k_{\text{B}}T}}\sim \frac{v_{w}(r)}{r}$$

for an ion of charge Z and mass *m*_*p*_*A* where *m*_*p*_ is the proton mass, plasma parameter ln Λ ≃ 20, and all other symbols have their usual meanings, this transition is expected to occur at a plasma density *n ~* 10^6^ cm^−3^, which corresponds to a radius r ~ 1.5 *R*_⊙_ where *v*_*w*_ ~ 100 km s^−1^ in the slow wind. In this region the density varies most strongly, and largely controls this transition. Obviously, ions of different elements will make this transition at various radii, leading to a much less “clean” transition than either of the first two. But this transition is crucially important to the wave-driven acceleration of the solar wind. Ions can only be accelerated into the solar wind by waves once they are decoupled from fluid motions (Cranmer et al. 1999; Miralles et al. 2001). Different ions will accelerate at different rates depending on where exactly they decouple and on how much MHD wave energy is available to them at their ion cyclotron resonant frequency or above their stochasticity threshold.

**Figure 1.**
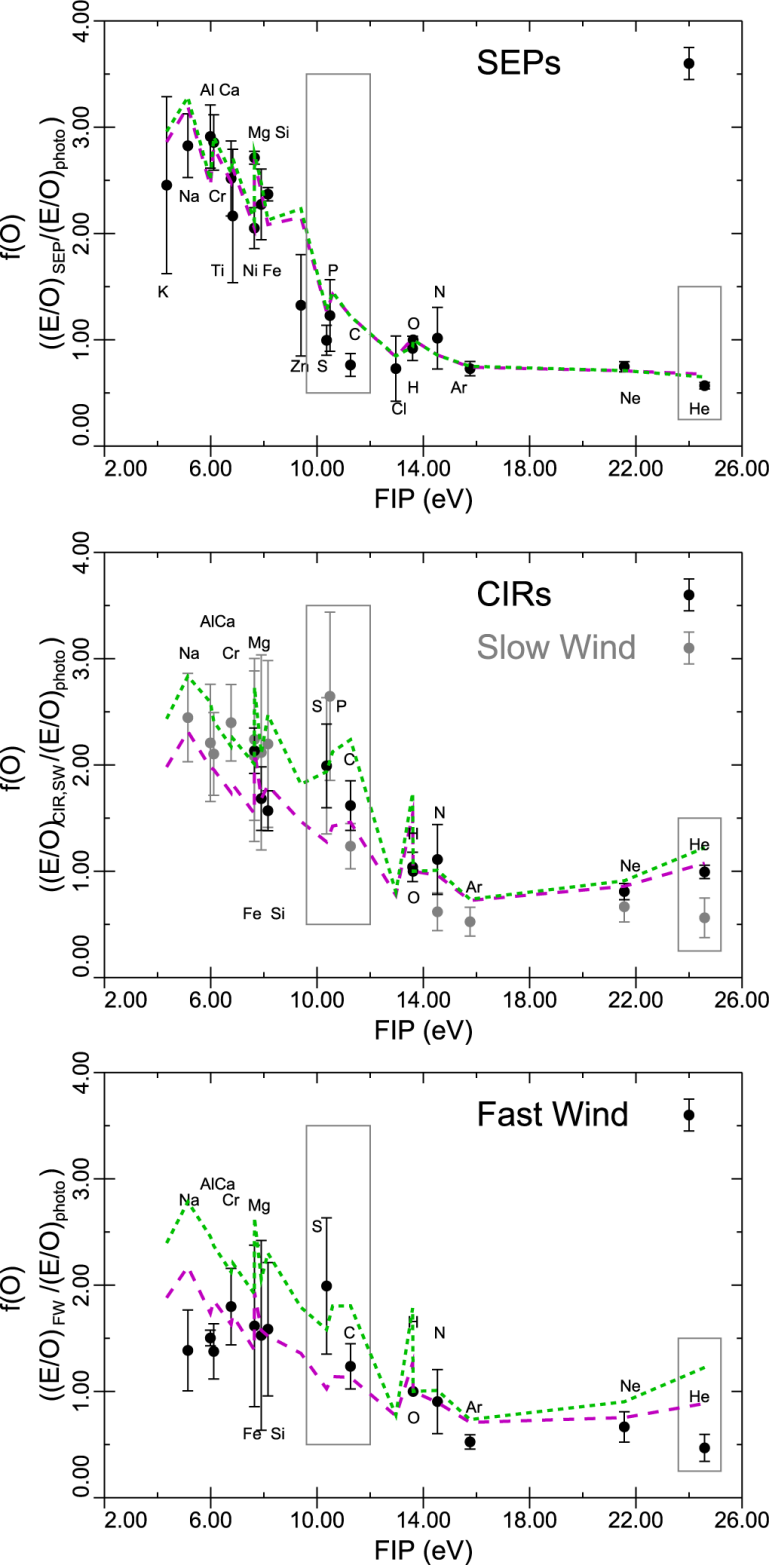
Variation of the first ionization potential (FIP) fractionation. Top: SEP fractionations relative to O shown as black circles with error bars from Table 1 in Reames (2018). Model calculations for a closed coronal loop are shown as a result of ponderomotive FIP fractionation by shear (magenta) and torsional (green) Alfvén waves and mass-dependent adiabatic invariant conservation (see Sections 2 and 3). Middle: corotating interaction region energetic particle fractionations (black circles; Reames 2018) and slow speed solar wind (dark gray; Bochsler 2009) relative to O. Models are for an open field with *B* = 30 G in the corona. Bottom: fast solar wind fractionations (Bochsler 2009) compared with open field models with coronal field of 10 G. Boxes highlight the S, P, C, and He fractionations that are especially variable.

The behavior of waves and how they interact with these three transition layers is crucial to the acceleration and elemental composition of the solar wind. The varieties of fractionation that are routinely exhibited by the solar corona and wind are shown in Figure 1, replotted from data given in Table 1 of Reames (2018). The top panel shows element fractionations for solar energetic particles (SEPs) relative to the O abundance of Caffau et al. (2011), given as black circles with error bars, together with a model calculation designed to match abundances determined remotely by spectroscopy of a closed coronal loop, with an assumed coronal magnetic field of 30 G for shear (magenta dashed curve) and torsional (green dashed curve) Alfvén waves. The calculation is described in detail below, but for now we point out that both in observations and the model, S, P, and C behave mainly as high-FIP elements, being fractionated by an insignificant amount. Reames (2018) points out that this correspondence in element abundances between SEPs and closed coronal loops means that the particles that end up being accelerated in shock waves must have an origin in the closed loop solar corona, and cannot be swept up out of the ambient solar wind, as previously argued elsewhere. Laming et al. (2013 and references therein) reach the same conclusion on somewhat different grounds.

The middle panel shows similar measurements for accelerated particles measured in corotating interaction regions (CIRs, black symbols) and slow solar wind (dark gray symbols). In contrast with the case above, in CIRs the accelerated particles are swept up directly from the solar wind, hence the correspondence between the sets of observations. The models show fractionations in open field with a magnetic field of 30 G at the top of the chromosphere for shear and torsional Alfvén waves as before. A key difference, picked up by both model curves but especially by the torsional Alfvén waves, is that S, P, and C now behave more like low-FIP elements. This difference in behavior between SEPs and solar wind had been visible previously in SEPs (Ko et al. 2013) and solar flares (Sylwester et al. 2008, 2012), compared with solar wind observations (Giammanco et al. 2007a, 2007b, 2008; Reisenfeld et al. 2007). It is also displayed in Figure 1 of Schmelz et al. (2012), and first commented, to our knowledge, by Rakowski & Laming (2012). As we argue further below, this difference in fractionation pattern is crucial to understanding slow solar wind origin, and the processes such as interchange reconnection that form it.

Finally, for completeness, in the bottom panel we show results for the fast solar wind, together with models (again for shear and torsional waves) for open field regions with coronal fields of and 10 G. With the exception of S which has large error bars, (but is measured lower by Gloeckler & Geiss 2007), shear Alfvén waves (the magenta curve) are clearly favored by the model (more details given in Section 2), while torsional waves better reproduce the slow solar wind abundances, especially S, P, and C.

Although interchange reconnection was originally introduced as a means of releasing FIP fractionated material in closed loops into the solar wind, we are finding that it is also important as a source of torsional Alfvén waves, which we discuss further below. Thus plasma fractionation on open field lines may be qualitatively different in coronal holes away from closed fields or in active regions close to closed field regions.

The differences between these panels suggest that possibilities exist for diagnosing the origin of the solar wind in terms of the magnetic geometry of the structure(s) from which it emanates in terms of the microphysics as embodied by the element abundances. In Section 2 of this paper, we give a more detailed discussion of the origins of the FIP fractionation and how the variations in fractionation may be related to wave properties in different magnetic structures. Section 3 gives model results, while Section 4 summarizes other possible mechanisms of fractionation. Section 5 outlines an observational approach to validate some of these hypotheses, and Section 6 concludes.

## 2. FIP Fractionation

### 2.1. Open versus Closed Field

**Figure 2.**
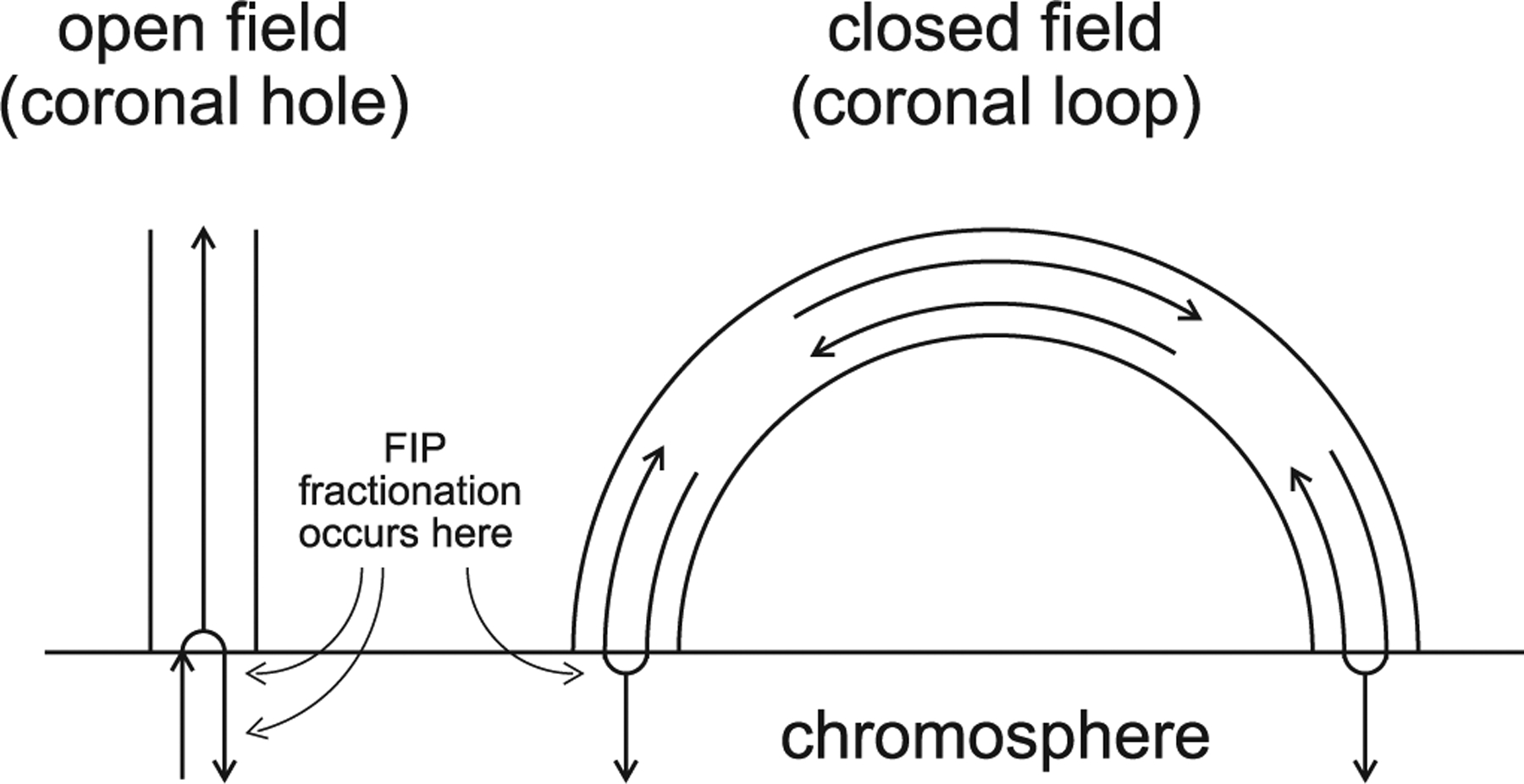
Schematic showing FIP fractionation in open and closed field regions. In the open field, waves impinge on footpoints from below but, in the closed field, wave generation within the coronal loop dominates.

We describe in more detail the calculations producing the model results in Figure 1. We begin with the two simple scenarios shown schematically in Figure 2, an open field region and a closed coronal loop, which serve as the basic models for fast solar wind and coronal or SEP abundances respectively. In open field regions, waves deriving from convection within the solar envelope propagate upward to the footpoint, and either enter the coronal hole or are reflected back down again. These waves entering the coronal hole ultimately drive the solar wind outflow. Typically the periods of these waves (three or five minutes) are too long for resonance with a closed coronal loop, and so in this case they are generally reflected back downward when encountering the footpoints of such loops. Resonant waves are most plausibly excited within the coronal loop itself, most likely as a byproduct of the mechanism(s) that heat the corona (Dahlburg et al. 2016; Tarr 2017). In open field regions, such a resonance does not exist, and only waves propagating up from footpoints are possible. Cranmer et al. (2007) was able to show that the MHD turbulence in an open slow solar wind flux tube could have some low-FIP abundance enhancement (i.e., the Fe/O ratio) without the need for closed loops to undergo interchange reconnection. Hence the slow solar wind composition is most likely a combination of the compositions arising from the two scenarios, as a closed loop interchange reconnects (e.g., Lynch et al. 2014; Higginson & Lynch 2018) with the neighboring open field to release its plasma into the solar wind. This has been recently discussed in terms of the evolution of the separatrix-web (S-Web; Antiochos et al. 2011), the network of quasi-separatrix layers formed by open field corridors within otherwise closed field regions.

Interchange reconnection is also important in exciting torsional Alfvén waves. Lynch et al. (2014) and Higginson & Lynch (2018) report simulations showing a large-scale torsional Alfvén wave arising as the open field reconnects with a twisted closed loop, with the twist being transferred to the resulting open field. The twist excites torsional waves as it relaxes. In the models presented above ponderomotive force from torsional waves seems to be important in fractionating S/O to the levels seen in the slow speed solar wind, and the torsional wave amplitudes seen in the simulation of Lynch et al. (2014) and in the observations of Tiwari et al. (2018) are consistent with our models. It is possible that this wave is not involved in the solar wind acceleration. Vasheghani Farahani et al. (2012) argue that these waves do not couple to other modes or to each other as well as shear Alfvén waves, meaning that any turbulent cascade will be less efficient in producing waves in the ion–cyclotron range that can resonate with solar wind ions. But conversely, they might better survive propagation through the chromosphere to fractionate the plasma. In this way the fast wind is also different; the (shear) waves that fractionate the plasma are also taken to be the waves that reflect, cascade, and ultimately accelerate the solar wind.

### 2.2. Ponderomotive Ion–Neutral Separation

Laming (2004) introduced the idea that ion–neutral separation in the chromosphere arises as a result of the ponderomotive force. This force arises as a result of Alfvén or fast-mode (collectively known as “Alfvénic” when close to parallel propagation) waves propagating through or reflecting from the solar chromosphere. In the absence of wave reflection or refraction (the Wentzel-Kramers-Brillouin approximation), the ponderomotive force is just the negative wave pressure gradient. However, in the presence of wave reflection or refraction, the wave particle interaction is mediated through the refractive index of the plasma, with the result that MHD waves and ions are attracted to each other (the opposite of the negative wave pressure gradient). A general form for the instantaneous ponderomotive acceleration, a, experienced by an ion is (see, e.g., the Appendix of Laming 2017)

(2) $$a=\frac{c^{2}}{2}\frac{\partial }{\partial z}\left(\frac{\delta E^{2}}{B^{2}}\right)$$

where *δE* is the wave electric field, *B* the ambient magnetic field, *c* the speed of light, and *z* is a coordinate along the magnetic field.

The element fractionation by the ponderomotive force is calculated from momentum equations for ions and neutrals in a background of protons and neutral hydrogen. The ratios, *f*_*k*_, of densities *ρ*_*k*_ for element k at upper and lower boundaries of the fractionation region *z*_*u*_ and *z*_*l*_ respectively, are given by the equation (Laming 2017)

(3) $$f_{k}=\frac{\rho_{k}\left(z_{u}\right)}{\rho_{k}\left(z_{l}\right)}\;=\text{exp}\left\{\int_{z_{l}}^{z_{u}}\frac{2\xi_{k}a\nu_{kn}/\left[\xi_{k}\nu_{kn}+\left(1-\xi_{k}\right)\nu_{ki}\right]}{2k_{\text{B}}T/m_{k}+v_{\|,\text{osc}}^{2}+2u_{k}^{2}}dz\right\},$$

where *ξ*_*k*_ is the element ionization fraction, *ν*_*ki*_ and *v*_*kn*_ are collision frequencies of ions and neutrals with the background gas (mainly hydrogen and protons, given by formulae in Laming 2004), $k_{\text{B}}T/m_{k}\left(=v_{z}^{2}\right)$ represents the square of the element thermal velocity along the z-direction, *u*_*k*_ is the upward flow speed, and *v*_∣∣,osc_ a longitudinal oscillatory speed, corresponding to upward- and downward-propagating sound waves. Because *ν*_*ki*_ ≫ *ν*_*kn*_ in the fractionation region at the top of the chromosphere, small departures of *ξ*_*k*_ from unity can result in large decreases in the fractionation. This feature is important in suppressing the fractionation of S, P, and C at the top of the chromosphere, while allowing it lower down where H is neutral, giving rise to the different fractionation of these elements in the various panels of Figure 1.

The specification of *v*_∣∣,osc_ is outlined in the next subsection. Here we describe the implementation of some important approximations near the plasma *β* = 1 layer. When *v*_∣∣,osc_ is greater than the local Alfvén speed, all fractionation is assumed to cease. We argue that the sound waves will excite counter-propagating Alfvén waves which can then cascade to microscopic scales, mixing the plasma at a rate much faster than it can be fractionated. In general, *v*_∣∣,osc_ has contributions from upward-propagating sound waves excited by solar convection, and sound wave excited in the chromosphere by the Alfvén wave driver (e.g., Arber et al. 2016) by the modulational instability (sometimes known as parametric excitation; e.g., Landau & Lifshitz 1976). Similar arguments restrict the fractionation to the plasma *β* < 1 region of the solar atmosphere. Here sound waves can also decay directly to counter-propagating Alfvén waves, which can again cause mixing after cascading to microscopic scales.

### 2.3. Chromospheric Model and Wave Fields

**Figure 3.**
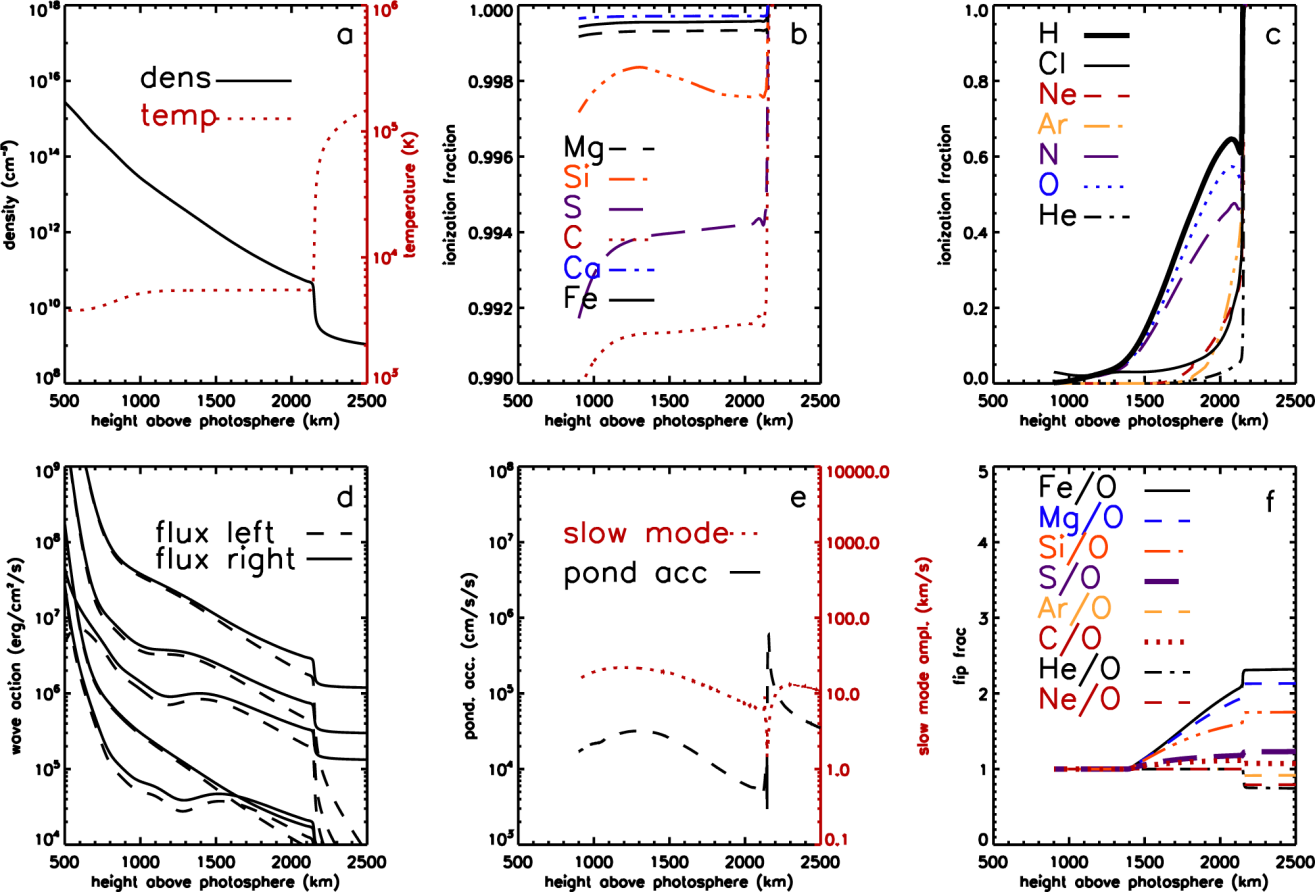
Chromospheric model for fast wind from an open field region. (a) Density and temperature structure of the chromosphere. (b, c) Chromospheric ionization fractions for low-FIP elements and high-FIP elements respectively. (d) Wave energy fluxes in each direction for the five waves in the open field model. (e) Ponderomotive acceleration (solid line) and the amplitude of slow mode waves induced by the Alfvén wave driver. (f) Fractionations resulting for selected elements relative to O, S, and C shown with thicker lines. Gas pressure and magnetic field pressure are equal at about 1000 km, magnetic field pressure dominating at higher altitudes.

We take the chromospheric model of Avrett & Loeser (2008) for temperature, density, and electron density profiles, combined with a force-free magnetic field calculated from formulae given by Athay (1981). This captures the behavior low in the chromosphere as the magnetic field decreases with height near the plasma *β* = 1 layer, and is constant with height above this region. The temperature and density profiles are shown in the top left panel of Figure 3. The hydrogen ionization balance dominating the electron density is shown as the thick line on the top right panel of Figure 3. The degree of ionization inferred observationally is higher than equilibrium at the local density and temperature would suggest, presumably due to the passage of shock waves that elevate the ionization fraction on timescales faster than that associated with electron– proton recombination (Carlsson & Stein 2002). Ionization balances for other elements are calculated here using the local temperature, density, and radiation field. This comprises coronal radiation from above, taken from Vernazza & Reeves (1978), absorbed progressively in the chromosphere, and trapped chromospheric Lyα photons. The coronal spectrum varies from coronal holes to active regions, introducing small variations in the ionization fraction of minor ions, most visible in the high-FIP elements. Atomic data are taken from Verner et al. (1996) for photoionization cross sections, and from Mazzotta et al. (1998) for collisional rates. We are only concerned with neutral atoms and singly charged ions so subsequent refinements to dielectronic recombination rates as considered in Bryans et al. (2009) are largely unimportant. We do, however, include the effects of electron density on the dielectronic recombination, following Nikolić et al. (2013). Uncertainties in the ionization balance are probably dominated by the underlying chromospheric model and the assumed coronal ionizing spectrum rather than by atomic data deficiencies.

Chromospheric acoustic waves are introduced to match simulations and data analysis in Heggland et al. (2011) and Carlsson et al. (2015). Acoustic waves with a flux of 10^8^ erg cm^−2^ s^−1^ propagate upwards through the chromosphere with their amplitude increasing as the density decreases in accordance with the WKB approximation, until the amplitude reaches the local sound speed. At this point we stop the amplitude growth, arguing that the excess energy is lost to the wave by radiation and conduction, principally cooling by Lyα with a timescale of the order of seconds. Lower down the cooling is dominated by H^−^ with a timescale of order of minutes (Ayres & Rabin 1996).

The Alfvén waves are modeled using transport equations given by Cranmer & van Ballegooijen (2005) and Laming (2015),

(4) $$\frac{\partial I_{\pm }}{\partial t}+\left(u\mp V_{\text{A}}\right)\frac{\partial I_{\pm }}{\partial z}=\left(u\pm V_{\text{A}}\right)\left(\frac{I_{\pm }}{4L}+\frac{I_{\mp }}{2L_{\text{A}}}\right)$$

where $I_{\pm }=\delta v\pm \delta B/\sqrt{4\pi \rho }$ are the Elsässer variables representing waves propagating in the ∓z-directions. The Alfvén wave spectrum in the coronal hole model is taken from from Cranmer et al. (2007). We specify five waves to match the peaks in the theoretical spectrum, and start the integration at an altitude of 500,000 km, where the outgoing waves dominate (Cranmer & van Ballegooijen 2005). At an altitude of 1000 km in the coronal hole, where the sound speed and Alfvén speeds are equal, the Alfvén wave solution corresponds to an energy flux of ~4 × 10^7^ erg cm^−2^ s^−1^, comparable to, but slightly less than, the upward acoustic wave energy flux that generates these waves.

In the closed field model, we assume coronal waves only, which are taken to be the loop resonant mode as in Laming (2012, 2017) and Rakowski & Laming (2012). The amplitude is adjusted to give a best match with observed FIP fractionations, and is typically ~50 km s^−1^. Simulations of coronal heating show that waves of this amplitude are indeed produced as a “by-product” of the heating mechanism (Dahlburg et al. 2016; Tarr 2017).

At high Alfvén wave energy fluxes, the ponderomotive force will modify the structure of the chromosphere itself. We estimate when this will occur as follows. The expression for the ponderomotive acceleration can be modified to (Laming 2015)

(5) $$a=\frac{a_{0}}{1+\left(\xi_{h}/4\right)\left(\nu_{\text{eff }}/\nu_{hi}\right)\left(\sum_{\text{waves}}\delta v^{2}/v_{h}^{2}\right)}$$

where *v*_eff_ = *v*_*hi*_*v*_*hn*_*/*(*ξ*_*h*_*v*_*hn*_ + (1 −*ξ*_*h*_)*v*_*hi*_) is the effective collision frequency of element h (in this case hydrogen) in terms of its collision frequencies when ionized *ν*_*hi*_, and when neutral *ν*_*hn*_, and $v_{h}^{2}=k_{\text{B}}T/m_{h}+v_{\|,\text{osc}}^{2}/2+u_{k}^{2}$ is the square of the hydrogen speed, in terms of its thermal speed, the amplitude of slow mode waves propagating through the chromosphere, and the flow speed in the chromospheric model. Since the ponderomotive force separates ions from neutrals, its effect on the background plasma to smooth out density gradients depends on the coupling between ionized and neutral hydrogen, and is strongest in regions where hydrogen is fully ionized (*ξ* = 1), and becomes significant at ponderomotive accelerations above about 10^6^ cm s^−2^, possibly giving rise to a mechanism of saturation.

## 3. Fractionation Model Results

### 3.1. Coronal Hole

Figure 3 shows the chromospheric portion of the solution of Equations (4) for a coronal hole. The magnetic field at the top of the chromosphere is 10 G, leading to a plasma *β* = 1 layer at an altitude of 1000 km above the photosphere. The top panels show the chromospheric density and temperature structure and the ionization balances for low and high FIP elements. The bottom three panels show the energy fluxes (left and right going) for the five shear Alfvén waves comprising the model spectrum, the ponderomotive acceleration and amplitude of sound waves generated by the Alfvén wave driver, and the resulting fractionations. At altitudes below about 1400 km, the amplitude of sound waves is higher than the local Alfvén speed and no fractionation occurs. Above this height, fractionation of Fe, Mg, and Si sets in with magnitude 1.5–2.0, S and C are much less enhanced, by 1.1–1.2, and Ar, Ne and He are depleted. This depletion of He is characteristic of fractionation concentrated at the top of the chromosphere.

### 3.2. Closed Coronal Loop

**Figure 4.**
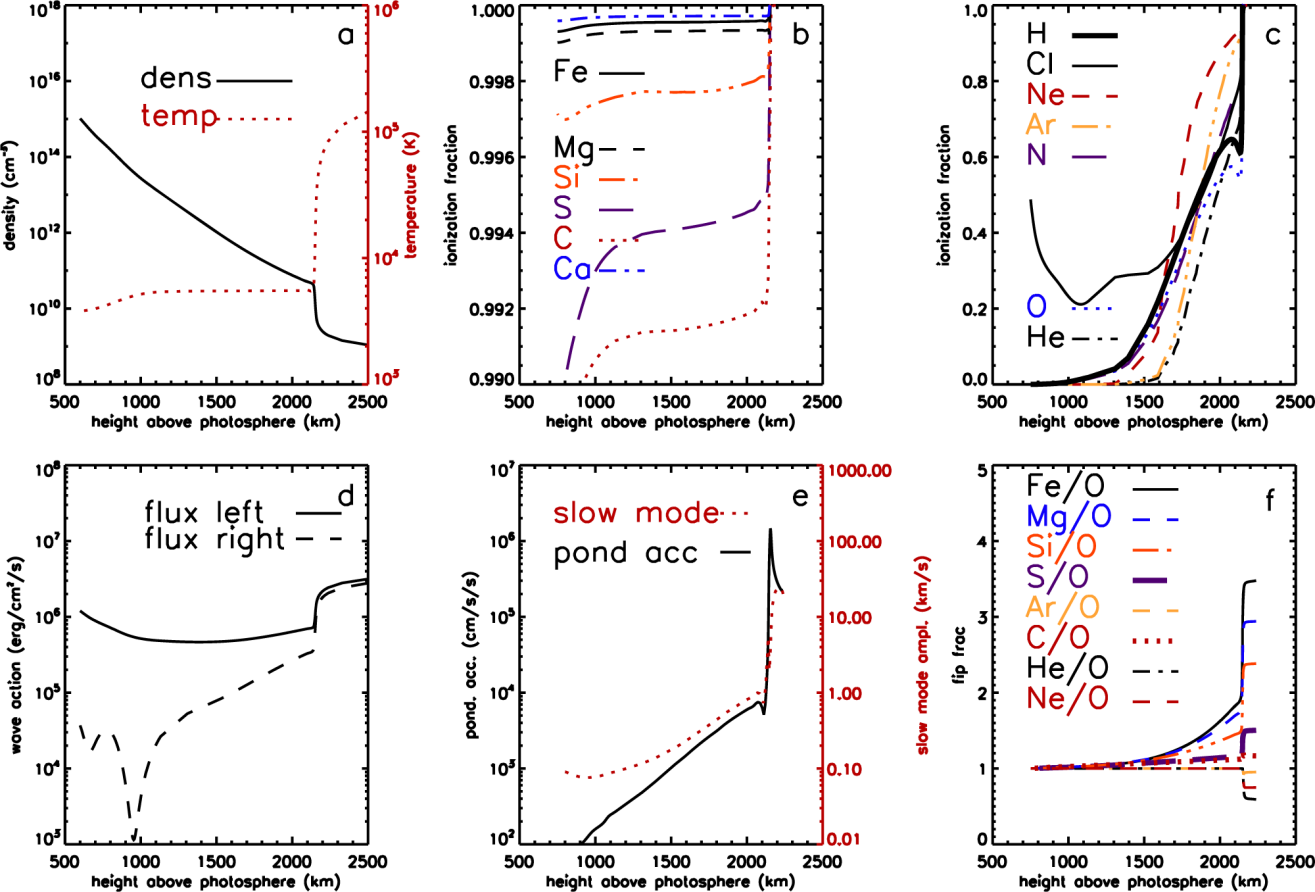
Chromospheric model for a closed field region. (a) Density and temperature structure of the chromosphere. (b, c) Chromospheric ionization fractions for low-FIP elements and high-FIP elements respectively. (d) Wave energy fluxes in each direction for the resonant wave in the closed field model. (e) Ponderomotive acceleration (solid line) and the amplitude of slow mode waves induced by the Alfvén wave driver. (f) Fractionations resulting for selected elements relative to O, S and C shown with thicker lines. Gas pressure and magnetic field pressure are equal at about 750 km, magnetic field pressure dominating at higher altitudes.

Figure 4 shows similar panels to Figure 3, but now for a closed coronal loop with a magnetic field at the top of the chromosphere of 30 G. The *β* = 1 layer is now at 750 km altitude. However, fractionation still is only significant at heights similar to that in the coronal hole. Here the reason is that the model contains only one coronal shear Alfvén wave that is trapped in the coronal loop, and insufficient wave energy leaks low enough in the chromosphere to cause fractionation lower down. The pattern of fractionation is similar to, but larger than, that in the coronal hole. Fe, Mg, and Si are enhanced by factors of 3–4.5, S and C by 1.1–1.6, and He and Ar are again depleted. Similarly to the coronal hole, the ponderomotive acceleration shows a spike of ~10^6^ cm s^−2^, about twice as large as in the coronal hole, at an altitude of 2150 km where the chromospheric density gradient is strongest, and this is where the strongest fractionation occurs.

### 3.3. Slow Speed Solar Wind

**Figure 5.**
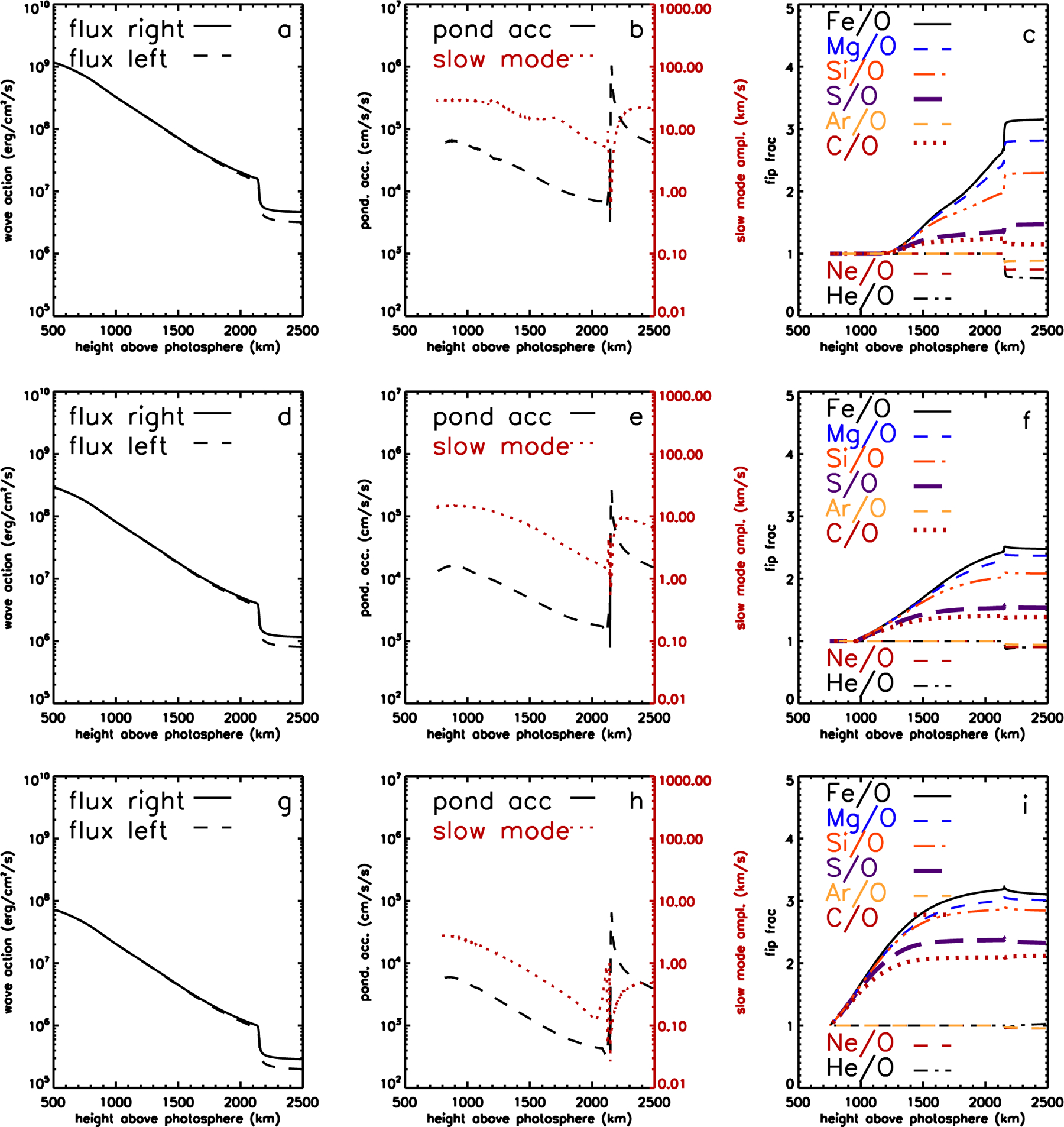
Chromospheric model for slow wind from an open field region showing the varieties of FIP fractionation expected. Top row: (a), (b), and (c) show wave energy fluxes, ponderomotive acceleration and slow mode wave amplitude, and fractionations for high shear wave energy fluxes. Strong FIP fractionation, depletion of He, and small enhancements of S and C result. Middle row: (d), (e), and (f) show the same plots for lower-amplitude shear Alfvén waves. Lower FIP fractionation, reduced He depletion, and stronger S and C enhancements are seen. Bottom row: (g), (h), and (i) show the same plots for torsional Alfvén waves. He depletion vanishes, and even stronger fractionation of S and C is exhibited.

Both examples above show S and C behaving more like high-FIP elements, in that they do not fractionate appreciably. Figure 5 shows examples of fractionation in the open field with a magnetic field similar to the closed loop (30 G at the top of the chromosphere) that show a transition from S and C behaving as high-FIP elements as above, to becoming fractionated like the low-FIP elements. The top row, (a), (b), and (c), show the (shear) Alfvén wave energy fluxes, and ponderomotive acceleration and associated slow mode amplitude, and the FIP fractionations respectively. This example has relatively high Alfvén wave amplitudes, leading to a spike in the ponderomotive acceleration of about 10^6^ cm s^−2^ as in the closed loop case, and similar fractionation to that case. The slow mode wave amplitude developing in the lower chromosphere is larger than the local Alfvén speed, suppressing any fractionation there.

The middle row, (d), (e), and (f), show similar plots, but for an open field with lower energy fluxes for the shear Alfvén waves. The significance of the spike in the ponderomotive acceleration is reduced, but slow modes lower down are of lower amplitude, allowing fractionation to occur there. The shift of FIP fractionation to lower altitudes where H is largely neutral allows S and C to behave more like low-FIP elements. In Equation (3) we no longer have *v*_*ki*_ >> *v*_*kn*_, and so a small departure of *ξ*_*k*_ from unity no longer suppresses the fractionation in the same way as it does in a background gas of protons. Hence elements like S, P, and C can become fractionated low in the chromosphere, whereas higher up they cannot. This behavior is even more pronounced in the bottom row, panels (g), (h), and (i), which show a similar model except that shear Alfvén waves have been replaced by torsional waves, which generate even lower slow mode wave amplitudes (Vasheghani Farahani et al. 2011; Laming 2017). Even more fractionation occurs close to the *β* = 1 layer, with correspondingly more S and C. Such waves, when combined with mass-dependent fractionation discussed above, give the green dashed curve in the middle panel of Figure 1. Note the enhanced S, P, and C compared to the magenta curve representing shear Alfvén waves. Such effects are much less prominent in the closed loop model. Here shear and torsional Alfvén waves produce essentially the same fractionation pattern (Laming 2017), because the Alfvén waves remain trapped in the loop and do not penetrate to the lower chromospheric regions where H is neutral, and where the amplitude of sound waves coming up from the photosphere is much lower.

When FIP fractionation is concentrated low in the chromosphere, He remains undepleted, and S, P, and C are fractionated. The reverse is true when FIP fractionation is concentrated at the top of the chromosphere: He and Ne are depleted, and S, P, and C are essentially unchanged. In this way the pattern of FIP fractionation can be seen to be dependent on wave properties in the solar atmosphere, and to offer an novel and unexpected window into this physics. In particular, perturbations akin to torsional Alfvén waves are expected as part of the slow solar wind release process through interchange reconnection. An open field reconnecting with a twisted closed field takes on the twist (e.g., Lynch et al. 2014; Higginson & Lynch 2018) which propagates away. This process should easily excite both upward- and downward-propagating torsional waves as it proceeds. Even so, this observed behavior may suppress the excitation of sound waves compared to shear waves even more than that modeled in Vasheghani Farahani et al. (2011) and Laming (2017), reinforcing our conclusion.

## 4. Mass- and Charge-dependent Fractionation and Acceleration

### 4.1. Introduction

While FIP fractionation by the ponderomotive force is the dominant mechanism of abundance modification, a number of other possibilities exist in the solar wind. Analysis of solar wind samples returned by the Genesis mission has revealed isotopic fractionation between fast and slow solar wind (Heber et al. 2012), where lighter isotopes are more abundant relative to heavy ones of the same element in the slow wind compared to the fast. This is the reverse of what Equation (3) would predict for the ponderomotive force, so clearly other mechanisms must be at work.

### 4.2. Inefficient Coulomb Drag

Inefficient Coulomb drag (ICD) is usually implemented following Geiss et al. (1970). Assuming that H flows fastest in the solar wind, the flow velocity of other elements *v*_*k*_ is calculated relative to that of H, *V*_H_, as

(6) $$v_{k}=V_{\text{H}}-\frac{3\sqrt{\pi }}{4}\frac{m_{p}}{4\pi e^{4}\text{ln}\Lambda }\frac{k_{\text{B}}T}{n}\sqrt{\frac{A+1}{A}\frac{2k_{\text{B}}T}{m_{p}}}\;\times \left(V_{\text{H}}\frac{dV_{\text{H}}}{dr}+\frac{GM_{⊙}}{r^{2}}\right)\left(\frac{2A-Z-1}{2Z^{2}}\right)\left(\frac{A+1}{A}\right).$$

An important point is that abundance modifications are only sustainable in the solar wind while there is a collisional connection back to the solar disk. Once the flow becomes collisionless according to Equation (1), no further fractionation is possible. All elements passing through this region must eventually flow out in the solar wind, and different elements become collisionless at different altitudes, giving

(7) $$v_{k}=V_{\text{H}}-\frac{r}{v_{w}(r)}\left(V_{\text{H}}\frac{dV_{\text{H}}}{dr}+\frac{GM_{⊙}}{r^{2}}\right)\left(\frac{2A-Z-1}{2A}\right),$$

with the implication that in the solar wind ICD only fractionates particles according to the variation of *r*/*v*_*w*_(*r*) where they freeze in, since the factor (2*A* − *Z* − 1)/2*A* varies much less from ion to ion than does (2*A* − *Z* −1)/2*Z*^2^ in Equation (6). At the time of writing, this is a difficult effect to quantify, but we speculate that it results in possibly a much smaller fractionation than quoted previously (e.g., Bodmer & Bochsler 1998; Bochsler 2007). The parameter controlling this most closely will be the plasma density, which has the strongest variation with *r*.

At 1 au, however, minor ions (including He) are generally observed to flow faster than H (e.g., Kohl et al. 2006; Berger et al. 2011), limiting the applicability of Equation (6). This preferential acceleration presumably sets in once the wind has become collisionless, where Equation (6) is no longer valid in any case.

### 4.3. Gravitational Settling

Gravitational settling in a closed coronal loop prior to eruption can be modeled with the same equation. The whole loop can be assumed to be collisionally coupled to the solar disk, so the complication from the transition to collisionless plasma does not arise. From the continuity equation

(8) $$\frac{\partial n_{k}}{\partial t}=-\nabla \cdot \left(n_{k}v_{k}\right)≃-2n_{k}v_{k}/L$$

where *L* is the loop length, and *v*_*k*_ is the element settling velocity (absolute magnitude) calculated from Equation (6) with *V*_H_ = 0. This has solutions of the form

(9) $$n_{k}\propto \text{exp}\left(-2v_{k}t/L\right).$$

Assuming *n* ~ 10^9^ cm^−3^, *T* ~ 10^6^ K, and *L* = 75,000 km, the gravitational settling (1/exp) times evaluate to 1.5, 3.6, and 5.0 days for He, O, and Ne, respectively. Thus such abundance modifications are only likely to occur in the most quiescent of solar coronal structures (see, e.g., Raymond et al. 1997). Landi & Testa (2015) observe variations in the Ne/O abundance ratio consistent with this, in quiescent coronal streamers and in the slowest speed solar wind at solar minimum of 2005–2008, with Ne/O increasing to 0.25 during this period, higher than its more usual value of 0.17. Kasper et al. (2012) and Rakowski & Laming (2012) observe the He/H and He/O abundance ratios moving in the opposite direction. While He depletion can be caused by the ponderomotive force as part of the FIP fractionation, Ne should behave similarly. And the He depletions observed, He/H as low as 1% (Kasper et al. 2012; Kepko et al. 2016), appear to be too extreme to be reproduced by the ponderomotive force, so gravitational settling where He settles relative to O, and O settles relative to Ne, appears to be the most plausible explanation. Finally, heavy ion dropouts are also observed on occasion in the solar wind (Weberg et al. 2012, 2015), clearly indicating gravitational settling prior to plasma release into the solar wind as in Feldman et al. (1998) where Fe (settling time according to the above of 2.4 days) is seen depleted relative to Si (4.3 days).

### 4.4. First Adiabatic Invariant Conservation

In Laming et al. (2017), it was argued that the dominant mass-dependent fractionation effect should be that of the conservation of the first adiabatic invariant, in conditions where the ion gyrofrequency Ω = *eB*/*m*_*k*_*c* ≫ *ν*_*ip*_. When an ion undergoes many gyro-orbits around the magnetic field line in the time between Coulomb collisions with other ions (mainly protons), the magnetic flux enclosed by its orbit is conserved. Hence $Br_{g}^{2}\propto v_{⊥}^{2}/B$ is constant (*r*_*g*_ is the particle gyroradius), giving rise to an acceleration

(10) $$\frac{dv_{z}}{dt}=-\frac{1}{2}\frac{dB}{dz}\frac{v_{⊥}^{2}}{B}$$

when $v^{2}=v_{z}^{2}+v_{⊥}^{2}$ is constant. While the plasma is still collisionally connected to the solar envelope (i.e., before it becomes collisionless and undergoes acceleration into the solar wind) a mass-dependent fractionation results:

(11) $$f_{a}=\text{exp}\left\{-\int \frac{dB/dz\left(v_{⊥}^{2}/B\right)}{2k_{\text{B}}T/m_{k}+v_{\|,\text{osc}}^{2}+2u_{k}^{2}}dz\right\}.$$

This arises because the thermal speeds $v_{⊥}^{2}$ and 2*k*_*B*_*T*/*m*_*k*_ are proportional to 1/*m*_*k*_, while $v_{\|,\text{osc}}^{2}$ and $u_{k}^{2}$ representing fluid motions are not, and are usually much larger, and can match the isotopic differences between high-speed and low-speed solar wind.

### 4.5. Resonant Heating by Ion Cyclotron Waves

Since the advent of the *SOlar and Heliospheric Observatory (SOHO*; Domingo et al. 1995) and ensuing imaging missions, the solar atmosphere has come to be increasingly appreciated as a dynamic and complex environment. Waves play a much larger role in shaping the plasma properties than hitherto assumed and they can have comparable energy densities to the thermal gas in the corona. For example, a major discovery made by the Ultraviolet Coronagraph-Spectrometer on SOHO (UVCS; Kohl et al. 1995, 1997, 2006) was that of significant heating in the O^5+^ ion inferred from spectral line broadening, beginning at altitudes where the plasma becomes collisionless according to Equation (1), and to a lesser extent also in Mg^9+^. It is likely that all heavy ions are heated in this manner and location; O VI and Mg X were the only ions accessible to UVCS observation with sufficient counting statistics. This heating is believed to derive from resonance with ion–cyclotron waves. Major questions surround the origin of the ion– cyclotron waves, with in situ generation, presumably via a turbulent cascade from lower-frequency Alfvén waves, being favored (Cranmer 2001; Hollweg & Isenberg 2002), and the degree of isotropy in the heating, with strongly anisotropic energization with perpendicular temperature, *T*_⊥_ ≫ *T*_||_, the parallel temperature, favored. This *T*_⊥_ is converted to parallel velocity in the expanding magnetic field lines by conservation of the first adiabatic invariant, leading to solar wind acceleration.

Further insight into these processes can only come from observing ion cyclotron resonant heating in a wider variety of ions, establishing the spectrum of ion cyclotron waves and the rates of acceleration of various ions into the solar wind. For example, ion energization can arise as ion cyclotron waves progressively cascade to higher frequencies, or are brought into resonance by frequency sweeping, and might be expected to lose all their energy to the lowest gyrofrequency ion in the plasma (e.g., Vocks & Marsch 2002). This would proceed until the velocity distribution function of that ion becomes sufficiently distorted to reach marginal stability, allowing the wave to pass through that resonance to the next lowest gyrofrequency ion. Such a case would have a quite different distribution of ion nonthermal line broadenings to a case where ion cyclotron waves were excited directly by, e.g., reconnection (Liu et al. 2011).

## 5. Observing Strategies to Test the Roles of Element Fractionation and MHD Waves

### 5.1. General Observing Concept

Off-limb observations give the best view of the solar corona uncontaminated by emission from plasma at lower altitudes. The choice of waveband is a tradeoff between count rates and the selection of diagnostic lines available, with the best compromise generally being found in the far-ultraviolet (FUV) and close by part of the extreme-ultraviolet (EUV) wavebands. Higher throughput may be achieved at longer wavelengths, especially from the ground (e.g., the Daniel K. Inouye Solar Telescope; Tritschler et al. 2015), but with reduced availability of useful diagnostic lines for our specific purposes. Pushing further into the EUV would give more useful lines, but with diminished count rates due to mirror and grating reflectivities. Additionally, the FUV/EUV combination includes the H Lyman series and also lines with radiative and collisionally excited components, adding to the diagnostic utility.

**Figure 6.**
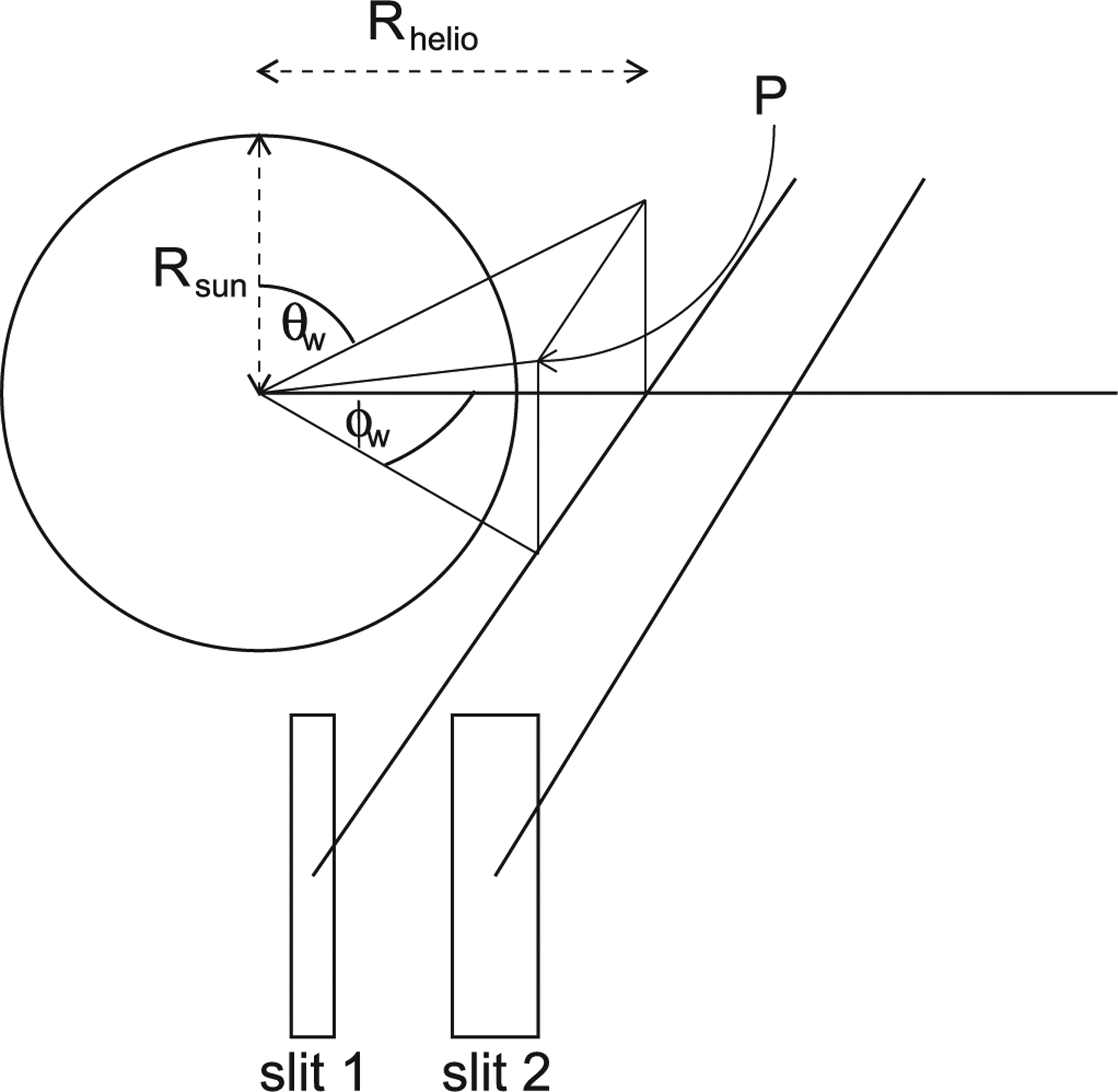
Schematic of off-limb observation geometry. In this example, two slits observe at heliocentric heights 1.5 and 2.2 *R*_⊙_. Radiation on an ion at point P at projected heliocentric radius Rhelio is calculated by integrating across the line profile, then over the solar disk, and finally along the line of sight.

Such off-limb UV spectroscopy would directly observe the element abundance fractionations (e.g., those illustrated in Table 1) in various coronal structures, allowing these to be traced back to the solar disk and related to the properties of MHD waves propagating in the solar atmosphere, with particular references to how these waves interact and drive the solar wind through ion–cyclotron resonance. This approach drives the spatial and temporal resolution of observations, discussed further below. Figure 6 shows a schematic diagram of the observation concept. Slits observing off limb at projected heliocentric distances between 1.3 and 3.0 *R*_⊙_ return EUV and FUV spectra. The slit heights are chosen to represent the solar corona fluid–kinetic transition region discussed above, where the acceleration of the solar wind commences, and a region where solar wind acceleration and the associated line broadening should be readily visible. Ideally, several slit configurations would be available, e.g., a single slit for detailed spectroscopy of the widest possible selection of lines, and two slits for observing only the strongest lines for wave and shock studies, allowing the discrimination between upward-and downward-propagating and standing waves.

In an alternative approach, the Spectral Investigation of the Coronal Environment instrument on the Solar Orbiter (Fludra et al. 2013) views the solar disk directly, in order to study the solar source of the wind simultaneously detected in situ on the same spacecraft. It will view a subset of the lines in our envisaged EUV bandpass, and use one slit (of varying sizes) at a time. These observations will be more focused on identifying the precise sources of the solar wind through their abundance patterns, and less on the wave physics and acceleration processes in the extended corona. They will, however, have strong S lines within their bandpass, allowing the study of some of the subtle fractionation issues discussed above.

In the following subsections, we consider the spectral bandpasses in the FUV and EUV that optimize the coverage of spectral lines from different low- and high-FIP elements for FIP fractionation studies (Section 5.2), and the special considerations required for lines that are also excited radiatively by absorption of light from the solar disk (Section 5.3). Following those, we discuss the observing approach for abundance studies, specifically He and S (Section 5.4), direct wave observations with two slits (Section 5.5), and the application of our strawman instrumentation to other topics in solar wind science (Section 5.6).

### 5.2. Spectral Bandpass and Resolution

In Tables 2 and 3 we give the spectral bandpasses (short and long in the UV range, with count rates appropriate to the quiet solar corona) most appropriate for testing the theoretical predictions above. They are similar to the UVCS bandpasses, but with extended wavelength ranges to observe a wider sample of coronal ions. The long-wavelength region has been extended to include the He II 1640 Å multiplet. Lines from S X and S XI appear in both the long- and short-wavelength range. These become very important since the only lines from carbon are C IV, which are difficult to compare with other similar temperature lines, and phosphorus has a low abundance, making its lines intrinsically weak. Feldman et al. (1997) identify the P IX 853.54 Å and 861.10 Å (2*s*^2^2*p*^3 4^*S*_3/2_–2*s*^2^2*p*^3 2^ *P*_3/2,1/2_), P XI 1307.57 Å and 1317.66 Å (2*s*^2^2*p*^3 4^*S*_3/2_–2*s*^2^2*p*^3 2^*D*_5/2,3/2_) and P XII 1096.71 Å (2*s*2*p*^1^*P*_1_–2*p*^2 1^*D*_2_) transitions, and Laming et al. (1996) gives calculations of the density dependence of the P IX 1317.66/1307.57 intensity ratio. Prominent lines from low-FIP ions Mg VII, VIII, IX, Si VII, VIII, IX, and Fe X, XI are available in the short-wavelength EUV bandpass, while the long-wavelength FUV region adds Mg IX and Fe XII, XIII. High-FIP ions are mainly available in the EUV; O VI, Ne VII, VIII, and Ar VIII, XII, with N V, O VII, and Ar XI also present in the FUV.

The primary science discussed in this paper, that of measuring relative element abundances as a means of understanding solar wind origins, does not strongly constrain the required resolution, since spectral line intensities are the main observables. Wave studies are more demanding in this respect. The H I Lyα line is typically 1 Å wide (e.g., Laming et al. 2013), which suggests a minimum resolution of *λ*/*δλ ~* 10^3^ (300 km s^−1^). The flux given in Table 3 with an effective area of ~1 cm^2^ gives a count rate of 30 s^−1^ in a 10 × 100 arcsec^2^ region of the corona, allowing the accumulation of ~1000 counts in 30 s. This in turn allows a determination of the line centroid to $\sim 1/\sqrt{1000}\sim 0.03Å$ or about 10 km s^−1^. Measurement of line profiles as a result of ion–cyclotron resonance heating will require still higher resolution, of order 3000 to resolve a 100 km s^−1^ line broadening.

### 5.3. Radiative Excitation

Also shown in Figure 6 is the geometry for computing the radiative excitation component of lines that are illuminated by disk radiation. For calculations where a detailed line profile is required, as in Laming et al. (2013), the radiatively excited component is calculated as a four-dimensional nested integral, integrating over the frequency overlap between the disk and coronal line profiles, the azimuthal and poloidal angles, *ϕ* and θ respectively, from point P projecting back to the solar disk, and finally the distance along the line of sight. The range for *ϕ* is where

(12) $$\phi_{w}-\sqrt{{\left(\text{arccos}\sqrt{1-1/R^{2}}\right)}^{2}+{\left(\theta_{w}-\theta \right)}^{2}}⩽\phi \;⩽\phi_{w}-\sqrt{{\left(\text{arccos}\sqrt{1-1/R^{2}}\right)}^{2}-{\left(\theta_{w}-\theta \right)}^{2}},$$

and for *θ*;

(13) $$\theta_{w}-\text{arccos}\sqrt{1-1/R^{2}}⩽\theta \;⩽\theta_{w}+\text{arccos}\sqrt{1-1/R^{2}},$$

where $R=R_{\text{helio }}\sqrt{{\text{sec}}^{2}\phi_{w}+{\text{cot}}^{2}\theta_{w}}$ is the heliocentric distance of point P. For applications where a detailed line profile is not required and predicted intensities are sufficient, and where the coronal ion distribution can be assumed isotropic and the solar illumination uniform, the integration over angles can be replaced by multiplying by the solid angle $2\pi (1-\sqrt{1-R_{⊙}^{2}/R^{2}})$. Auchere (2005) investigated relaxing both of these idealizations, and Raouafi & Solanki (2004) considered deviations from radial flow in the solar wind caused by the super-radial expansion of magnetic field lines, though further discussion is beyond our scope here.

**Figure 7.**
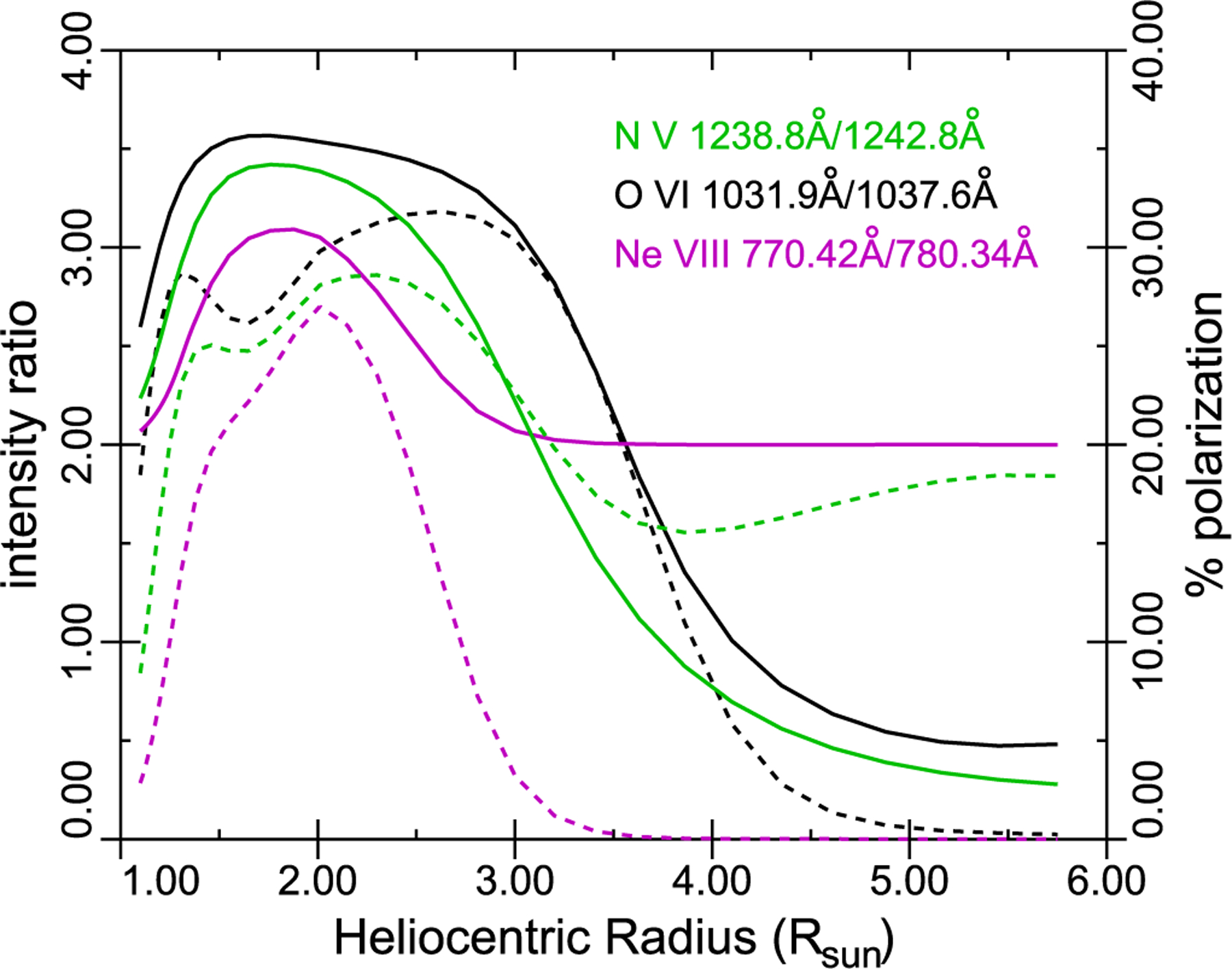
N V, O VI, and Ne VIII line intensity ratios as a function of heliocentric radius, showing the intensity ratio (solid) and the polarization resulting from radiative excitation (dash), to be read on the right-hand axis.

**Figure 8.**
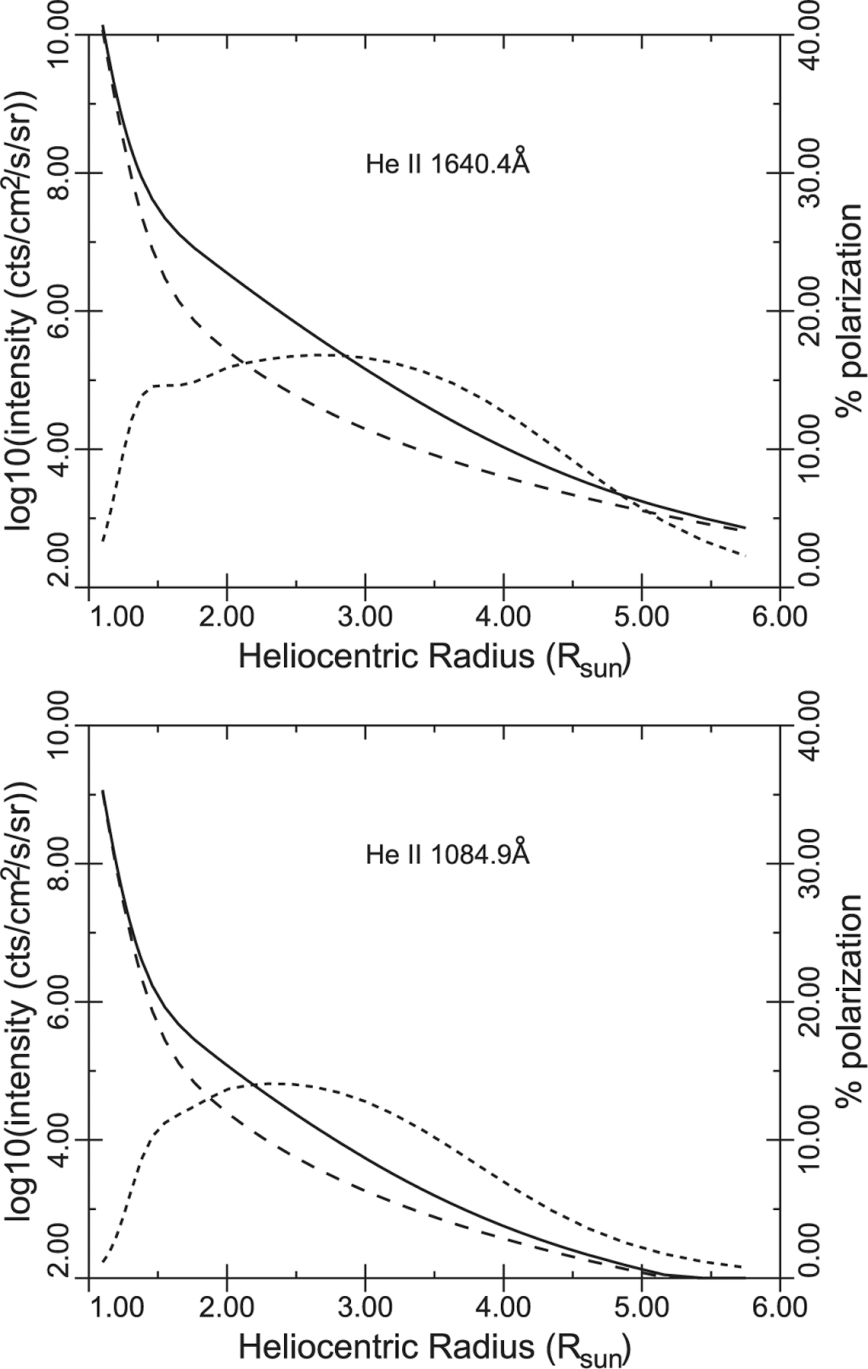
He II line intensities as a function of heliocentric radius, showing total (solid) and collisionally excited (long dash) components. The radiatively excited component (not shown separately) is polarized, giving overall line polarization shown by the short dash line to be read on the right-hand axis.

This last approximation is used in calculating the radiative and collisional components of the Li-like doublets, N V, O VI, and Ne VIII shown in Figure 7, and of the He II 1640.4 and 1084.9 Å multiplets, shown in Figure 8, both for quiet Sun conditions. In Figure 7, the solid curves give the intensity ratio between the two components of the doublet. In conditions of pure collisional excitation, this is precisely 2. Radiative excitation favors the stronger of the two components, so the intensity rises above 2 as we move off-limb, with a theoretical maximum in conditions of pure radiative excitation of 4. Above about 3–4 *R*_⊙_, the coronal lines are Doppler shifted out of resonance with the disk emission (known as “Doppler dimming”), and the intensity ratio returns to 2, unless other lines exist in the disk spectrum (e.g., Fe XII 1242.007 Å in the case of N V and C II 1036.34/1037.02 Å for O VI; see Li et al. 1998) which move into resonance to continue the radiative excitation. The short dashed lines show the (negative, i.e., orthogonal to the radial direction) polarization in the stronger component of the doublet, arising due to the radiative excitation (e.g., Cranmer 1998).

In Figure 8 in each case the solid line gives the total He II line intensity. The long dash lines give the contribution from collisional excitation, the remainder being radiatively excited by emission from the solar disk in the 1*s*–3*p* 256.37 Å and the 1*s*–5*p* 237.36 Å lines for 1640.4 and 1084.9 Å respectively. These last processes are calculated using line intensities observed by Malinovsky & Heroux (1973) and Linsky et al. (1976), with line widths inferred from Brown et al. (2008). Close to the solar limb collisional excitation dominates. As the electron density declines moving out into the corona, collisional excitation proportional to density squared declines faster than radiative excitation, and radiative excitation dominates. Even further out, beyond 3–4 *R*_⊙_ in this example, the acceleration of the solar wind has Doppler shifted the coronal line profile out of coincidence with the disk emission, and collisional excitation is again important. Radiative excitation leads to linear polarization in the line, given by the short dash curve to be read on the right-hand axis.

The polarization has two effects on the measured line intensity, compared to the unpolarized case. The first is that the polarized light is emitted anisotropically. This is included in the calculation using the redistribution functions given by Cranmer (1998). The second effect arises if the instrumentation has polarization sensitivity, which needs to be corrected for. In the usual case, to be discussed further below, using gratings near normal incidence (the grating is the dominant polarization-sensitive component), we estimate a polarization sensitivity of only a few percent which, when observing a line polarized to 10%–20%, leads to errors in the intensity measurement of order 1%. This is well below other uncertainties (mainly counting statistics), and so is considered negligible from here on. A future experiment to measure the polarization in the He II lines could be directly interpreted in terms of the acceleration of the He component of the solar wind.

The coronal He abundance (Laming & Feldman 2001, 2003) is also a key diagnostic of solar wind acceleration. Rakowski & Laming (2012) showed that He abundance variations also result from the ponderomotive force that generates the FIP fractionation. Kasper et al. (2007, 2012) found extreme He abundance variations in the slowest speed solar wind near solar minimum. There is a complex interplay between the heating, acceleration, and wave absorption by helium (e.g., Kasper et al. 2008; Bourouaine et al. 2011, 2013; Chandran et al. 2013; Verscharen et al. 2013) and direct observation of such complex interplay will provide strong confirmation of wave driving of the solar wind.

### 5.4. Abundances and Waves

The discussion above suggests that coronal sources of the slow speed solar wind may be detectable by their abundance signature(s). Table 1 summarizes the fractionations expected for fast wind, closed coronal loop, and slow wind (i.e., open field, but with similar magnetic field to the closed loop), in each case for ponderomotive fractionation alone, and for a combination of ponderomotive and adiabatic invariant conservation designed to reproduce the isotopic fractionation seen in Genesis sample return data. While the basic FIP fractionation can be similar between material originating in closed loops or in open field regions, subtle details like the fractionation of S, P, and C, and also He and Ne, can vary. This is potentially an important diagnostic. The slow solar wind is believed to originate in interchange reconnection between closed and open fields, and the released wind should have have a composition determined by the relative amounts of originally closed and open field plasma that ultimately are released. The S abundance measurements of Giammanco et al. (2007a, 2007b) suggest that this is indeed the case, falling as they do between our closed loop and open field slow solar wind models. We therefore expect He, C, Ne, P, and S, the elements that change the most between the closed loop and slow wind models in Table 1, to be the most important element abundances to study.

To estimate the potential instrument performance, let us consider the requirement for detecting a factor of two change in the S or He abundance. The most intense lines of S are the S X 1196.217 and 1212.932 Å lines in the FUV bandpass, where the effective area is usually highest. Assuming a 10″ slit at 1.5 *R*_⊙_, the solar wind is moving at 20 km s^−1^ and takes 375 s to cross the slit field of view. Taking 100 counts (very conservative) in each line as a minimum to detect a factor of two change, and an effective area of 0.2 cm^2^, this can be done in 375 s within a solid angle of 3.7 × 10^−9^ rad, or approximately 12 5 × 12 5. The He II 1640.4 Å multiplet is less intense, and falls in a region of lower throughput, leading to about an order of magnitude less signal, or meaningful abundance measurements when integrated over 10″ × 100″ solid angle. At 2 *R*_⊙_ heliocentric distance, the line intensities are two orders of magnitude lower. The effective area, however, could be higher and a wider slit would lead to about one order of magnitude higher count rate.

### 5.5. Direct Wave Observations with Two Slits

The different regimes of the solar wind are known to be distinguishable by their turbulence and wave characteristics (e.g., Bruno & Carbone 2013; Ko et al. 2018). The fast wind shows mainly Alfvénic, but unbalanced, turbulence, while the slow wind is more balanced, but less Alfvénic. These characteristics match naively with the thought that FIP fractionated slow wind originates in closed coronal loops, where the balanced turbulence is a relic of that trapped in the loop, while relatively unfractionated fast wind originates from open field where, with Alfvén waves propagating up from the chromosphere, the turbulence is naturally unbalanced.

However the solar wind is not so simple. Interchange reconnection between open and closed fields is necessary to allow the plasma originally contained in loops to escape (e.g., Antiochos et al. 2011). And as we have shown above (Section 2.6), the high S abundance in the slow speed solar wind appears to require nonresonant waves, most plausibly from an open field region. The double-slit geometry outlined above would allow us to make direct observations of waves, and assess their frequency, wavenumber, mode, cross-helicity, etc.

Table 4 summarizes the observational properties of the various MHD wave modes, and how they might be identified from variations in line centroid, width, and intensity. Simultaneous observations in two slits also allows inferences on direction of motion and cross-helicity (i.e., degree to which waves are “balanced”). Consider two counterpropagating waves with amplitudes *a* ad *b*,

(14) a exp i(ωt−kz)+b exp i(ωt+kz)=2b exp iωt cos kz+(a−b) exp i(ωt−kz).

With the first slit at *z* = 0, the signal is ∝(*a* + *b*) exp *iωt* but at a second slit a projected distance L away, the signal is 2*b* exp *iωt* cos *kL* + (*a* − *b*) exp *i*(*ωt* − *kL*). The balanced portion of the disturbance produces oscillations is in phase in both slits. The unbalanced portion produces a second oscillation in the second slit with phase difference −*ikL*. Except when *kL* = 2*π*, balanced and unbalanced waves can be diagnosed, for comparison with predictions coming from the abundance pattern.

Low-frequency coronal waves themselves will be thus revealed by careful observation of the central region of the Lyα line profile. Recently, five minute Alfvénic waves have been detected with the Coronal Multichannel Polarimeter (Tomczyk et al. 2007) at low heights where the plasma is collisional, together with Alfvénic turbulence in coronal loops (De Moortel et al. 2014; Liu et al. 2014), and in an open field (Morton et al. 2015), albeit with lower amplitudes than expected. McIntosh & De Pontieu (2012) discuss possible reasons for this, e.g., the “dilution” of the signal by foreground and background emission. It is also possible that waves exist with higher amplitudes at different frequencies, as yet undetected. Mancuso & Raymond (2015) detect propagating kink waves (an almost parallel propagating fast-mode wave) with SOHO/UVCS revealed by Doppler shift oscillations in H I Ly*α*.

### 5.6. Shock Waves

Coronal mass ejections (CMEs) can also drive waves through the solar corona with important consequences for SEP acceleration when these steepen into shocks as the magnetic field decreases off-limb. Two-slit observations can determine the height of shock formation and the plasma properties of the pre-CME corona (Raymond et al. 2003). It is important to correlate the He abundance of the pre-CME corona with the large variations of He abundance in SEPs. Limits on the Alfvén and shock speeds (key parameters in SEP acceleration models) can be set by detection of the shock arrival at different heights as determined by the timing of the increase in line widths of UV emission lines (Mancuso et al. 2003; Ciaravella et al. 2006). The angle between the shock front and the magnetic field requires the pre-shock field direction, which can be determined from streamer morphology.

Shocks are seen in white light images because they compress the plasma (Vourlidas et al. 2003, 2013; Liu et al. 2017). They appear in UV spectra as drastic increases in line widths due to shock heating (Mancuso et al. 2002; Vourlidas & Bemporad 2012). These observations provide the compression ratio in the shock (a key parameter determining SEP spectral shape, e.g., Kwon & Vourlidas 2018) and information about thermal equilibration among electrons, protons, and ions (Bemporad et al. 2014). At high effective area, a large number of ionization states will be available for observation with this instrument concept, revealing the progress of ionization behind the shock consistently and providing the electron temperature (Ma et al. 2011). UV spectroscopy can test collisionless theories of multiion shock heating as a function of mass-per-charge (Lee & Wu 2000; Zimbardo 2011). Polarimetry, if available, can also yield inferences on shock microphysics (Shimoda et al. 2018), following the initial prediction (Laming 1990) and discovery (Sparks et al. 2015) of polarized emission in H*α* from collisionless shock waves in SN 1006. In the solar case, H I Lyα, usually polarized in a north–south direction due to resonant scattering of disk radiation, will be Doppler shifted out of resonance with the disk line and become polarized in a direction along the shock velocity vector, usually close to east– west, by collisions with the anisotropic post-shock electron and proton distributions.

Spectroscopy can also shed light on the heating and acceleration of CMEs. UVCS observations of Fe^17+^ (*T*_*e*_ ≃ 6 MK) within thin structures trailing CMEs provide evidence for reconnection in current sheets, a key prediction of many CME initiation models (Ciaravella et al. 2002; Lin et al. 2015). Other UVCS measurements show that the thermal energy is comparable to or can even exceed the CME kinetic energy (Akmal et al. 2001; Murphy et al. 2011). Yet these observations are too few and far between to allow a detailed investigation of the energy transfer in eruptive events. An instrument concept with greatly increased sensitivity over the UVCS telescope will observe many high-temperature (multiple Fe ions from 18+ to 21+) and low-temperature lines (C 3+, Si 3+), which can greatly expand our understanding of the CME initiation and initial evolution.

## 6. Conclusions

Our emerging understanding of FIP fractionation in terms of the ponderomotive force due to Alfvén waves, and improved observations revealing hitherto unexpected variations in the abundances of He, S, P, and C, suggest that we are on the cusp of significant breakthroughs in solar wind science. The S, P, C abundance enhancements can be traced to the differing altitudes in the chromosphere at which fractionation occurs, and this in turn can be traced to the differing properties of the Alfvén waves causing the fractionation, with respect to the magnetic structures in which they are propagating. Relationships should exist between the solar wind abundances and the nature of the turbulence entrained within it, a prime example being the cross-helicity or degree of balance between sunward and anti-sunward propagating waves. The cross-helicity is a crucial parameter in the development of a turbulent cascade, by means of which fluctuations on large scales can be transferred to smaller and smaller scales until they resonate with solar ion winds, heating and ultimately accelerating them.

Multi-slit off-limb spectroscopy in the EUV and FUV thus holds great promise for discoveries in solar wind science. Following on from the pioneering observations of SOHO/UVCS, with modern fabrication techniques we expect an increase of over a factor of 100 in instrument sensitivity, greatly extending the range of detectable spectral lines and the height off-limb at which observations can be made. Extending the UVCS bandpass to include the He II 1640 Å multiplet will capture He abundance variations, as well as S and C. Solar wind acceleration is one of the phenomena associated with the transition from fluid to collisionless plasma, and it offers a probe of this third transition layer in the solar atmosphere.

## Figures and Tables

**Table 1:** Model Corona and Wind Fractionations

Element	Closed	Loop	Slow	Wind	Fast	Wind
H	0.81	1.01	1.39	1.74	1.01	1.27
He	0.57	0.68	1.03	1.22	0.74	0.89
C	1.16	1.22	2.12	2.24	1.07	1.13
N	0.84	0.86	0.98	1.016	0.87	0.90
Ne	0.75	0.71	0.96	0.91	0.79	0.75
Na	3.48	3.20	3.09	2.84	2.37	2.117
Mg	2.96	2.68	3.01	2.73	2.13	1.93
Al	2.79	2.45	2.95	2.59	1.98	1.73
Si	2.40	2.08	2.84	2.47	1.75	1.52
P	1.70	1.43	2.52	2.12	1.36	1.14
S	1.52	1.26	2.33	1.94	1.23	1.02
Cl	1.04	0.84	1.00	0.80	0.95	0.77
Ar	0.96	0.74	0.95	0.74	0.92	0.71
K	3.67	2.86	3.13	2.44	2.42	1.88
Ca	3.64	2.81	3.12	2.41	2.40	1.85
Ti	3.62	2.61	3.12	2.25	2.38	1.71
Cr	3.55	2.48	3.11	2.17	2.33	1.63
Fe	3.52	2.39	3.11	2.11	2.32	1.58
Ni	3.09	2.05	3.02	2.01	2.09	1.39
Zn	3.37	2.15	2.85	1.824	2.13	1.36

**Note**. All fractionations given relative to O. The first column for each model gives ponderomotive fractionation, and the second column gives ponderomotive and adiabatic invariant conservation combined, as shown in Figure 1. The slow wind model assumes torsional Alfvén waves.

**Table 2:** Spectral Lines for Quiet Corona and Wind Fractionations, Short Wavelength

Wavelength	UVCS / SUMER	CHIANTI	Ion	Transition
700.24	5.5e7		Ar VIII	3*s* ^2^*S*_1/2_–3*p* ^2^*P*_3/2_
703.63	1.6e7		A1 IX	2*s*^2^2*p* ^2^*P*_3/2_–2*s*2*p*^2 4^*P*_3/2_
706.05	5.7e8		Mg IX	2*s*^2 1^*S*_0_–2*s*2*p* ^3^*P*_1_
713.81	2.5e7		Ar VIII	3*s* ^2^*S*_1/2_–3*p* ^2^*P*_1/2_
749.55	8.8e7		Mg IX	2*s*2*p* ^1^*P*_1_–2*p*^2 1^*D*_2_
770.42	2.2e9	6.6e9	Ne VIII	2*s* ^2^*S*_1/2_−2*p* ^2^*P*_3/2_
772.29	1.0e8		Mg VIII	2*s*^2^2*p* ^2^*P*_3/2_–2*s*2*p*^2 4^*P*_5/2_
772.53	6.0e6		A1 VIII	2*s*^2^2*p*^2 3^*P*_2_*-*2*s*2*p*^3 5^*S*_2_
776.25	1.70e8		SX	2*p*^3 4^*S*_3/2_–2*p*^3 2^*P*_3/2_
780.34	1.1e9	3.3e9	Ne VIII	2*s* ^2^*S*_1/2_–2*p* ^2^*P*_1/2_
782.37	8.0e7		Mg VIII	2*s*^2^2*p*^2^*P*_3/2_–2*s*2*p*^2 4^*P*_3/2_
782.96	3.0e7		S XI	2*p*^2 3^*P*1–2*p*^2 1^*S*_0_
789.44	0.9e7		Mg VIII	2*s*^2^2*p* ^2^*P*_3/2_–2*s*2*p*^2 4^*P*_1/2_
789.78	0.4e7		Na VIII	2*s*^2 1^*S*_0_–2*s*2*p* ^3^*P*_1_
854.66	0.8e7		Mg VII	2*s*^2^2*p*^2 3^*P*_1_–2*s*2*p*^3 5^*S*_2_
868.11	1.2e7		Mg VII	2*p*^2 3^*P*_2_–2*p*^3 5^*S*_2_
895.16	1.2e8	3.6e8	Ne VII	2*s*^2 1^*S*_0_–2*s*2*p* ^3^*P*_1_
944.37	1.6e8	4.8e8	Si VIII	2*p*^3 4^*S*_3/2_–2*p*^3 2^*P*_3/2_
949.24	5.4e7	1.5e8	Si VIII	2p^3 4^*S*_3/2_–2*p*^3 2^*P*_1/2_
950.16	8.3e7	2.5e8	Si IX	2*p*^2 3^*P*1–2*p*^2 1^*S*_0_
972.54	2.4e8		H I	1*s* ^2^*S*_1/2_–4*p* ^2^*P*_1/2,3/2_
1005.541	5.5e5	2.02e7	Si VII	2*s*^2^2*p*^3^ (^2^*P*)3*s* ^3^*P*_1_–2*s*^2^2*p*^3^ (^2^*P*)3*p* ^3^*P*_2_
1009.908		7.77e7	Si VII	2*s*^2^2*p*^3^ (^2^*P*)3*s*^3^ *P*_2_–2*s*^2^2*p*^3^ (^2^*P*)3*p* ^3^*P*_2_
1018.60	2.5e7		Ar XII	2*s*^2^2*p*^3 4^ *S*_3/2_–2*s*^2^2*p*^3 2^ *D*_5/2_
1018.903		4.64e7	Fe XI	3*s*^2^3*p*^3^ (^4^*S*)3*d* 5*D*_3_–3*s*^2^3*p*^3^ (^2^*P*)3*d* ^3^*F*_4_
1025.724	1.2e9	5.30e8	H I	1*s* ^2^*S*_1/2_–3*p* ^2^*P*_1/2,3/2_
1028.026	5.0e7	8.94e8	Fe X	3*s*^2^3*p*^4^ (^3^*P*)3*d* ^4^*D*_7/2_–3*s*^2^3*p*^4^ (^1^*D*)3*d* ^2^*F*_7/2_
1028.957	1.5e8	2.47e8	Fe XI	3*s*^2^3*p*^3^ (^4^*S*)3*d* ^5^*D*_4_–3*s*^2^3*p*^3^ (^2^*P*)3*d* ^3^*F*_4_
1031.914	4.0e9	2.96e10	O VI	l*s*^2^2*s* ^2^*S*_1/2_–1*s*^2^2*p* ^2^*P*_3/2_
1037.615	1.3e9	1.48e10	O VI	l*s*^2^2*s* ^2^*S*_1/2_–1*s*^2^2*p* ^2^*P*_1/2_
1049.155	7.0e6	9.38e7	Si VII	2*s*^2^2*p*^4 3^*P*_1_–2*s*^2^2*p*^4 1^*S*_0_
1051.538	1.0e6	1.00e7	S VII	2*p*^5^3*s*^3^*P*_2_–2*p*^5^3*p*^3^*S*_1_
1053.998	3.0e6	2.85e7	A1 VII	2*s*^2^2*p*^3 4^*S*_3/2_–2*s*^2^2*p*^3 2^ *P*_3/2_
1054.87	2.5e7		Ar XII	2*s*^2^2*p*^3 4^*S*_3/2_–2*s*^2^2*p*^3 2^ *D*_3/2_
1056.917	1.0e6	1.14e7	A1 VII	2*s*^2^2*p*^3 4^*S*_3/2_–2*s*^2^2*p*^3 2^*P*_1/2_
1084.9		1.65e7	He II	2–5
1132.774	4.0e6	3.94e7	Si VII	2*s*^2^2*p*^3^ (^2^*D*)3*s* ^3^*D*_3_–2*s*^2^2*p*^3^ (^2^*D*)3*p* ^3^*F*_4_
1135.353	1.6e7	5.46e7	Si VII	2*s*^2^2*p*^3^ (^4^*S*)3*s* ^5^*S*_2_–2*s*^2^2*p*^3^ (^4^*S*)3*p* ^5^*P*_3_
1137.240	2.0e6	1.57e7	Si VII	2*s*^2^2*p*^3^ (^2^*D*)3*s* ^3^*D*_2_–2*s*^2^2*p*^3^ (^2^*D*)3*p* ^3^*F*_3_
1146.528	2.0e6	1.84e7	Si VII	2*s*^2^2*p*^3^ (^4^*S*)3*s* ^5^*S*_2_–2*s*^2^2*p*^3^ (^4^*S*)3*p* ^5^*P*_1_
1167.775	2.4e6	3.76e7	Si VII	2*s*^2^2*p*^3^ (^4^*S*)3*s* ^3^*S*_1_–2*s*^2^2*p*^3^ (^4^*S*)3*p* ^3^*P*_2_
1182.455		2.62e7	Si VIII	2*s*^2^2*p*^2^ (^3^*P*)3*s* ^4^*P*_1/2_–2*s*^2^2*p*^2^ (^3^*P*)3*p* ^4^*D*_3/2_
1183.995	5.0e6	6.13e7	Si VIII	2*s*^2^2*p*^2^ (^3^P)3*s* ^4^*P*_3/2_–2*s*^2^2*p*^2^ (^3^*P*)3*p* ^4^*D*_5/2_
1189.487	1.4e7	1.34e8	Si VIII	2*s*^2^2*p*^2^ (^3^*P*)3*s* ^4^*P*_5/2_–2*s*^2^2*p*^2^ (^3^*P*)3*p* ^4^*D*_7/2_
1189.867	6.2e6	6.72e7	Mg VII	2*s*^2^2*p*^2 3^3 *P*_1_–2*s*^2^2*p*^2 1^*S*_0_

**Note**. Intensities are in photons cm^−2^ s^−1^ sr^−1^ computed from CHIANTI with a synthetic DEM matching that of the quiet Sun for log T ⩾ 6.0 with density = 10^7^ cm^−3^. This is divided by 1000 to match UVCS observations at 1.4 *R*_⊙_.

**Table 3:** Spectral Lines for Quiet Corona and Wind Fractionations, Long Wavelength

Wavelength	UVCS / SUMER	CHIANTI	Ion	Transition
1196.217	2.5e8	3.61e8	S X	2*s*^2^2*p*^3 4^*S*_3/2_–2*s*^2^2*p*^3 2^*D*_5/2_
1212.932	5.0e8	3.35e8	S X	2*s*^2^2*p*^3 4^*S*_3/2_–2*s*^2^2*p*^3 2^*D*_3/2_
1215.670		1.79e11	H I	1*s* ^2^*S*_1/2_–2*p* ^2^*p*_1/2,3/2_
1216.399		2.04e7	Si VIII	2*s*^2^2*p*^2^ (^3^*P*)3*s* ^4^*P*_3/2_–2*s*^2^2*p*^2^ (^3^*P*)3*p* ^4^*D*_3/2_
1216.430		2.33e8	Fe XIII	3*s*^2^3*p*^2 3^*P*_1_–3*s*^2^3*p*^2 1^*S*_0_
1238.823	3.0e8	8.70e8	N V	1*s*^2^2*s* ^2^*S*_1/2_–1*s*^2^2*p* ^2^*P*_3/2_
1242.007	1.0e9	9.25e8	Fe XII	3*s*^2^3*p*^3 4^ *S*_3/2_–3*s*^2^3*p*^3 2^ *P*_3/2_
1242.806	1.5e8	4.36e8	N V	1*s*^2^2*s* ^2^*S*_1/2_–1*s*^2^2*p* ^2^*P*_1/2_
1327.316		1.24e7	Mg VII	2*s*^2^2*p*3*s* ^3^*P*_1_–2*s*^2^2*p*3*p* ^3^*P*_0_
1334.223		1.53e7	Mg VII	2*s*^2^2*p*3*s* ^3^*P*_2_–2*s*^2^2*p*3*p* ^3^*P*_2_
1349.403	5.0e8	5.88e8	Fe XII	3*s*^2^3*p*^3 4^ *S*_3/2_–3*s*^2^3*p*^3 2^ *P*_1/2_
1350.439		1.24e7	Mg VII	2*s*^2^2*p*3*s* ^3^*P*_2_–2*s*^2^2*p*3*p* ^3^*P*_1_
1392.098	1.7e8	1.25e7	Ar XI	2*s*^2^2*p*^4 3^*P*_2_–2*s*^2^2*p*^4 1^*D*_2_
1409.446	3.0e7	1.05e8	Fe XI	3*s*^2^3*p*^3^ (^4^*S*)3*d* ^5^*D*_3_–3*s*^2^3*p*^3^ (^2^*D*)3*d* ^1^*G*_4_
1428.758	1.5e8	4.36e8	Fe XI	3*s*^2^3*p*^3^ (^4^*S*)3*d* ^5^*D*_4_–3*s*^2^3*p*^3^ (^2^*D*)3*d* ^1^*G*_4_
1440.510	2.9e7	2.53e9	Si VIII	2*s*^2^2*p*^3 4^ *S*_3/2_–2*s*^2^2*p*^3 2^ *D*_5/2_
1445.737	3.6e8	4.60e9	Si VIII	2*s*^2^2*p*^3 4^ *S*_3/2_–2*s*^2^2*p*^3 2^ *D*_3/2_
1463.489	1.0e8	7.85e8	Fe X	3*s*^2^3*p*^4^ (^3^*P*)3*d* ^4^*F*_9/2_–3*s*^2^3*p*^4^ (^1^*D*)3*d* ^2^*F*_7/2_
1467.070	1.3e8	2.19e9	Fe XI	3*s*^2^3*p*^4 3^*P*_1_–3*s*^2^3*p*^4 1^*S*_0_
1510.508		2.87e7	Ni XI	3*s*^2^3*p*^5^3*d* ^3^*P*_1_–3*s*^2^3*p*^5^3*d* ^3^*D*_2_
1537.282		2.30e7	Mg IX	2*s*3*s* ^3^*S*_1_–2*s*3*p* ^3^*P*_2_
1548.189		4.48e8	C IV	1*s*^2^2*s* ^2^*S*_1/2_–1*s*^2^2*p* ^2^*P*_3/2_
1550.775		2.24e8	C IV	1*s*^2^2*s* ^2^*S*_1/2_–1*s*^2^2*p* ^2^*P*_1/2_
1582.557		3.30e8	Fe XI	3*s*^2^3*p*^3^ (^4^*S*)3*d* ^5^*D*_4_–3*s*^2^3*p*^3^ (^2^*D*)3*d* ^3^*G*_5_
1603.209		5.85e8	Fe X	3*s*^2^3*p*^4^ (^3^*P*)3*d* ^4^*D*_7/2_–3*s*^2^3*p*^4^ (^1^*D*)3*d* ^2^*G*_7/2_
1603.351		2.81e8	Fe X	3*s*^2^3*p*^4^ (^3^*P*)3*d* ^4^*D*_5/2_–3*s*^2^3*p*^4^ (^1^*D*)3*d* ^2^*G*_7/2_
1604.779		5.93e7	A1 VII	2*s*^2^2*p*^3 4^ *S*_3/2_–2*s*^2^2*p*^3 2^ *D*_3/2_
1605.938		6.06e7	Ni XI	3*s*^2^3*p*^5^3*d* ^3^*P*_2_–3*s*^2^3*p*^5^3*d* ^1^*F*_3_
1611.710		2.03e8	Fe X	3*s*^2^3*p*^4^ (^3^*P*)3*d* ^4^*D*_7/2_–3*s*^2^3*p*^4^ (^1^*D*)3*d* ^2^*G*_9/2_
1614.390		7.03e7	Fe XI	3*s*^2^3*p*^3^ (^4^*S*)3*d* ^5^*D*_3_–3*s*^2^3*p*^3^ (^2^*D*)3*d* ^3^*G*_4_
1614.495		2.09e7	S XI	2*s*^2^2*p*^2 3^*P*_1_–2*s*^2^2*p*^2 1^*D*_2_
1623.609		7.51e7	O VII	1*s*2*s* ^3^*S*_1_–1*s*2*p* ^3^*P*_2_
1639.777		6.24e8	Fe XI	3*s*^2^3*p*^3^ (^4^*S*)3*d* ^5^*D*_4_–3*s*^2^3*p*^3^ (^2^*D*)3*d* ^3^*G*_4_
1639.861		1.49e7	O VII	1*s*2*s* ^3^*S*_1_–1*s*2*p* ^3^*P*_0_
1640.40		1.40e8	He II	2–3

**Note**. Intensities are in photons cm^−2^ s^−1^ sr^−1^ computed from CHIANTI with a synthetic DEM matching that of the quiet Sun for log T ⩾ 6.0 with density = 10^7^ cm^−3^. This is divided by 1000 to match UVCS observations at 1.4 *R*_⊙_.

**Table 4:** Wave Modes Determined by Correlations between Oscillations of *δ*I, *δ*W, and *δ*λ

	Line Shift *δλ* = 0		Line Shift *δλ* ≠ 0	
	Line width *δW* = 0	Line width *δW* ≠ 0	Line width *δW* = 0	Line width *δW* ≠ 0
Intensity *δI* = 0	Shear or torsional Alfvén wave with p.o.s. oscillation (*k* along l.o.s)	Unresolved torsional Alfvén with *k* in p.o.s.	Shear Alfvén wave with *k* in p.o.s.	(Partially) resolved torsional Alfvén wave with *k* in p.o.s.
Intensity *δI* ≠ 0	No wave	Sound wave or fast mode with oscillation in p.o.s.	No wave	Sound wave or fast mode with oscillation along l.o.s.

**Note**. p.o.s. = plane of sky; l.o.s. = line of sight.
